# A zebrafish model of Ifih1-driven Aicardi–Goutières syndrome reproduces the interferon signature and the exacerbated inflammation of patients

**DOI:** 10.3389/fimmu.2023.1294766

**Published:** 2023-11-24

**Authors:** Beatriz Bernal-Bermúdez, Alicia Martínez-López, Francisco J. Martínez-Morcillo, Sylwia D. Tyrkalska, Teresa Martínez-Menchón, Pablo Mesa-del-Castillo, María L. Cayuela, Victoriano Mulero, Diana García-Moreno

**Affiliations:** ^1^ Departamento de Biología Celular e Histología, Facultad de Biología, Universidad de Murcia, Murcia, Spain; ^2^ Instituto Murciano de Investigación Biosanitaria (IMIB)-Pascual Parrilla, Murcia, Spain; ^3^ Centro de Investigación Biomédica en Red de Enfermedades Raras (CIBERER), Instituto de Salud Carlos III, Madrid, Spain; ^4^ Hospital Clínico Universitario Virgen de la Arrixaca, Murcia, Spain

**Keywords:** type I IFN, IFIH1, zebrafish avatar, autoimmunity, drug screening

## Abstract

Type I interferonopathies are a heterogenic group of rare diseases associated with an increase in type I interferon (IFN). The main challenge for the study of Type I interferonopathies is the lack of a well-founded animal model to better characterize the phenotype as well as to perform fast and large drug screenings to offer the best treatment options. In this study, we report the development of a transgenic zebrafish model of Type I interferonopathy overexpressing *ifih1* carrying the mutation p.Arg742His (*Tg(ifih1_mut)*), corresponding to the human mutation p.Arg779His. RNA sequence analysis from *Tg(ifih1_mut)* larvae revealed a systemic inflammation and IFN signature upon a suboptimal poly I:C induction compared with wild-type larvae, confirming the phenotype observed in patients suffering from Type I interferonopathies. More interestingly, the phenotype was manifested in the zebrafish inflammation and Type I IFN reporters *nfkb:eGFP* and *isg15:eGFP*, respectively, making this zebrafish model suitable for future high-throughput chemical screening (HTS). Using the unique advantages of the zebrafish model for gene editing, we have generated *Tg(ifih1_mut)* knocked down for *mavs* and *ikbke*, which completely abrogated the Poly I:C induction and activation of the GFP of the reporters. Finally, we used an FDA-approved drug, Baricitinib (Jak1/Jak2 inhibitor), which was able to reduce the inflammation and the ISG expression. Our results demonstrate the potential of this model to further understand AGS pathological mechanisms and to identify novel therapeutic drugs by HTS.

## Introduction

The Interferon (IFN) Induced Helicase C domain 1 gene *IFIH1* gene encodes the Melanoma Differentiation–Associated protein 5 (MDA5), which belongs to the RIG-I-Like Receptors (RLRs) family, a group of cytosolic receptors that recognize dsRNA. RLR receptors are in charge of the recognition of the viral RNA, and this receptor–ligand interaction activates the signaling cascade through the adaptor protein Mitochondrial Anti-Viral Signaling (MAVS)/Inhibitor of Nuclear factor κB Kinase subunit Epsilon (IKBKE), which elicits the translocation of the transcription factors IFN Regulatory Factor 3 (IRF3) and Nuclear Factor κB (NF-κB) to the nucleus, which activate the transcription of proinflammatory cytokines and IFN-β, that, in turn, induce the production of IFN-Stimulated Genes (ISGs) through the interaction with the IFN-α/β receptor, which transduce the intracellular signal through Janus Kinase 2 (JAK)/Signal Transducer and Activator of Transcription STAT (1).

Gain-of-function mutations in the human *IFIH1* result in the development of human type I interferonopathies, which are characterized by constitutive upregulation of type I IFN (2, 3). This particularity makes the *IFIH1* gene together with RLRs responsible for the development of the Singleton-Merten syndrome (SMS) (4, 5), an autosomal dominant disease that is manifested by abnormalities in the correct development of the skeleton and vascular system, the appearance of calcifications in the aorta and mitral valve, muscle weakness, psoriasis, and recurrent infections (6–8). Moreover, mutations in *IFIH1* have also been included in the list of genes responsible for the development of the autoimmune Aicardi–Goutières syndrome (AGS), a mimic of congenital infection manifested by an increased production of type I interferon (IFN-α) in blood. In humans, the AGS phenotype is associated with mutations in eight other different genes, namely, *TREX1* (9), *RNASEH2A*, *RNASEH2B*, *RNASEH2C* (10), *SAMHD1* (11), *ADAR1* (12), *LSM11* (13), and *RNU7-1* (14), involved either in nucleic acid metabolism or in the constitutive activation of their sensors, which, in turn, activates the cellular innate IFN-α-mediated immune response (9, 15).

The phenotypic manifestations of AGS are diverse and present different clinical symptoms even among patients with gain-of-function mutations in *IFIH1*. In fact, in the case of patients bearing *IFIH1* mutations, it is common to develop clinical symptoms that overlap between AGS and SMS (16), and the reasons are still not fully understood. It has been suggested that the differences in the penetrance of the immunological disease as well as in the appearance of the different phenotypes could be driven by the existence of other genetical or environmental factors (17). The only common feature that is always shared by both syndromes is the upregulation of the type I IFN.

With the aim of studying the mechanism of disease of AGS and SMS patients, there have been several attempts to develop animal models that reproduce the patient phenotypes, most of them using mice, that have helped in the understanding of these syndromes. In a very recent study, Ohto et al. (18) generated transgenic mice expressing human MDA5-R779H and observed that these mice developed systemic upregulation of type I IFN, myocarditis, and lupus-like nephritis, but no signs of brain calcification or chilblain lesions. Two other works (3, 19) show that mice expressing MDA5-G821S spontaneously developed lupus-like nephritis, encephalitis, upregulation of type I IFN, and SMS-like skeletal abnormalities. Finally, Emralino et al. (20) also show that mice expressing human MDA5-R822Q developed SMS-like heart fibrosis, aortic valve enlargement and calcification, and activation of a systemic ISG signature.

Another essential aspect of developing animal models for AGS or SMS is to use them as a tool to test and find new treatments for these diseases. In this matter, mouse models present clear disadvantages, as they do not allow the test of large-scale drug screenings, limited by the big amount of animals needed, their short life span when bearing particular mutations (3, 19, 20), or the time-consuming routes of administration (gavage, intramuscular, intraperitoneal, or intravenous injections). In this work, we present a zebrafish model that expresses an *ifih1* mutation p.Arg742His (corresponding to the human p.Arg779His) that develops the ISGs signature and the inflammation phenotype typically observed in AGS patients. Moreover, pharmacological inhibition of Jak and genetic inhibition of either *mavs* or *ikbk*e abrogated the overexpression of ISGs and inflammatory cytokines, which can be easily assayed using the IFN and inflammation zebrafish reporter lines *isg15:eGFP* and *nfkb:eGFP*, respectively. Our results demonstrate the usefulness of this model to further understand AGS pathological mechanisms and to identify novel therapeutic drugs by high-throughput chemical screening (HTS).

## Results

### Poly I:C stimulation is required to trigger type I IFN production in larvae overexpressing p.Arg742His Ifih1

**Figure 1:**
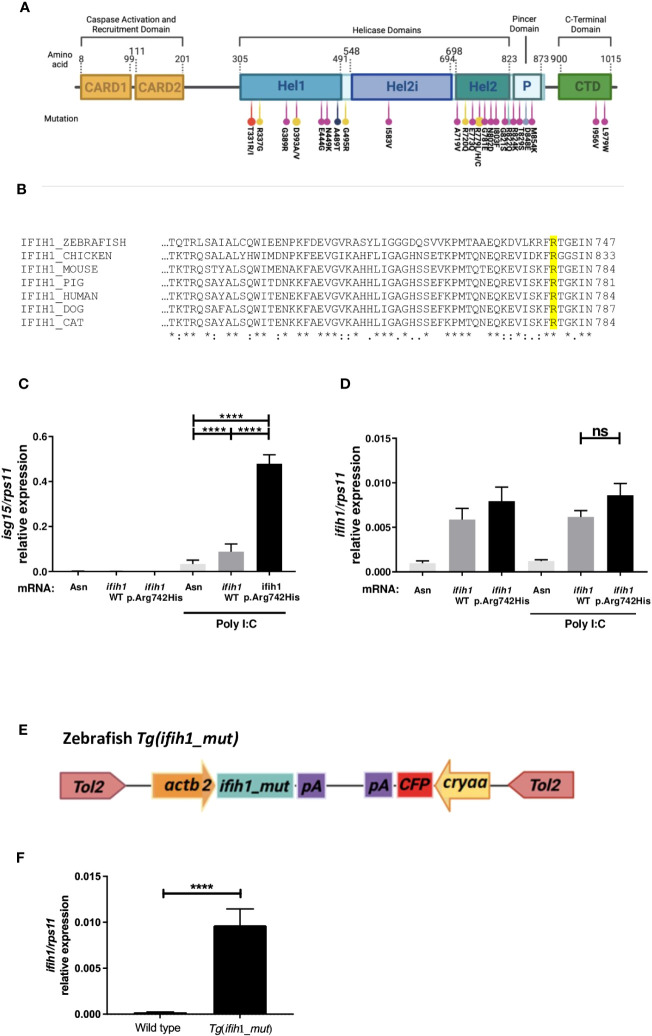
Localization, sequence alignments, *ifih1* mRNA overexpression, and *Tg*(*ifih1_mut)* line design. **(A)** Protein diagram showing the positions of protein domains and their amino acid boundaries within the 1,025 residues of IFIH1 (MDA5) protein. The 28 previously published mutations are annotated. CARD, caspase activation recruitment domain; Hel, helicase domain, where Hel1 and Hel2 are the two conserved core helicase domains and Hel2i is an insertion domain that is conserved in the RIG-I–like helicase family; P, pincer or bridge region connecting Hel2 to the C-terminal domain (CTD) involved in binding double-stranded RNA. Red (24), yellow (2), purple (22), black (16), blue (4), and light purple (23). **(B)** Protein sequence alignment of IFIH1 from different species; IFIH1_ZEBRAFISH (ENSDART00000003913.10), IFIH1_CHICKEN (ENSGALT00010050768.1), IFIH1_MOUSE (ENSMUST00000028259.12), IFH1_PIG (ENSSSCT00000061806.3), IFIH1_HUMAN (ENST00000649979.2), IFH1_DOG (ENSCAFT00845053824.1), IFIH1_CAT (ENSFCAT00000026735.4). **(C, D)** RT-qPCR of *isg15* **(C)** or *ifih1* **(D)** from 3-dpf zebrafish larvae injected with wild-type (WT) or mutant (p.Arg742His) *ifih1* mRNA with or without 25 pg/egg of poly I:C. **(E)** Scheme showing zebrafish p.Arg742His transgenic (Tg) design *Tg(ifih1_mut)* and **(F)** expression of *ifih1* in the transgenic line *Tg(ifih1_mut)* by RT-PCR. p-values were calculated using one-way ANOVA and Tukey multiple range test. ns, not significant; ****p ≤ 0.0001.

To date, 28 pathogenic variants have already been described for the *IFIH1* gene (2–4, 16, 19–24), all of them being monoallelic missense mutations either transmitted or generated *de novo* (**Figure 1A**). Interestingly, most of them are localized in the MDA5 helicase domain, which is a highly conserved domain between species (**Figure 1B**), but they can also be found in the pincer region (P) connecting Hel2 to the C-terminal domain (CTD) and in the same CTD that is implicated in de dsRNA binding. We chose the human mutation p.Arg779His, as it has been reported that it is able to upregulate type I IFN in mice (18) and our main aim is to develop a zebrafish model of AGS to find new drugs that can abrogate IFN overexpression. As the Arginine-779 amino acid in the Hel2 region is conserved in zebrafish, we overexpressed the zebrafish mutation variant *ifih1* p.Arg742His, corresponding to the amino acid position in the zebrafish sequence (**Figure 1B**).

To find out if the overexpression of either wild-type *ifih1* or *ifih1* mutant p.Arg742His in zebrafish larvae displayed the type I IFN phenotype, zebrafish eggs were microinjected with mRNA encoding the wild type *ifih1* or *ifih1* mutant p.Arg742His. As seen in **Figures 1C**, **D**, neither the wild-type *ifih1* nor the mutant were able to increase the mRNA levels of *isg15*, assayed by RT-qPCR, unless poly I:C was also injected. Although the overexpression of the wild-type *ifih1* did also increase the transcript levels of *isg15* compared to control mRNA (Asn, antisense control mRNA) when injected with poly I:C, the mutant *ifih1* p.Arg742His triggered the strongest induction of *isg15*, suggesting that this mutation increases the affinity of zebrafish *ifih1* towards the poly I:C, as it has been shown in human (2). Of note, a suboptimal dose of 25 pg/egg of poly I:C, which was unable to induce a strong *isg15* in wild-type fish compared with the mutant *ifih1* p.Arg742His, was used in all experiments (**Figure S1**).

This result prompted us to generate a transgenic zebrafish that stably and ubiquitously expressed the *ifih1* p.Arg742His mutant. To achieve this, we used a Tol2 transposon construct bearing the promoter from the housekeeping gene actin, beta 2 (*actb2*) driven by the expression of *ifih1* p.Arg742His and a Cyan Fluorescent Protein (CFP) eye-specific (crystallin, alpha A, and *cryaa*) transgenesis marker in the opposite orientation, which we named *Tg(ifih1_mut)* (**Figure 1E**). The expression of *ifih1* was measured by RT-PCR in the *Tg(ifih1_mut)* line (**Figure 1F**). This approach eliminates the expression variability associated to mRNA injection experiments. This line showed normal development, life expectancy, and Mendelian ratio (data not shown).

### Zebrafish Tg(ifih1_mut) displays systemic inflammation and IFN signature upon poly I:C induction

**Figure 2:**
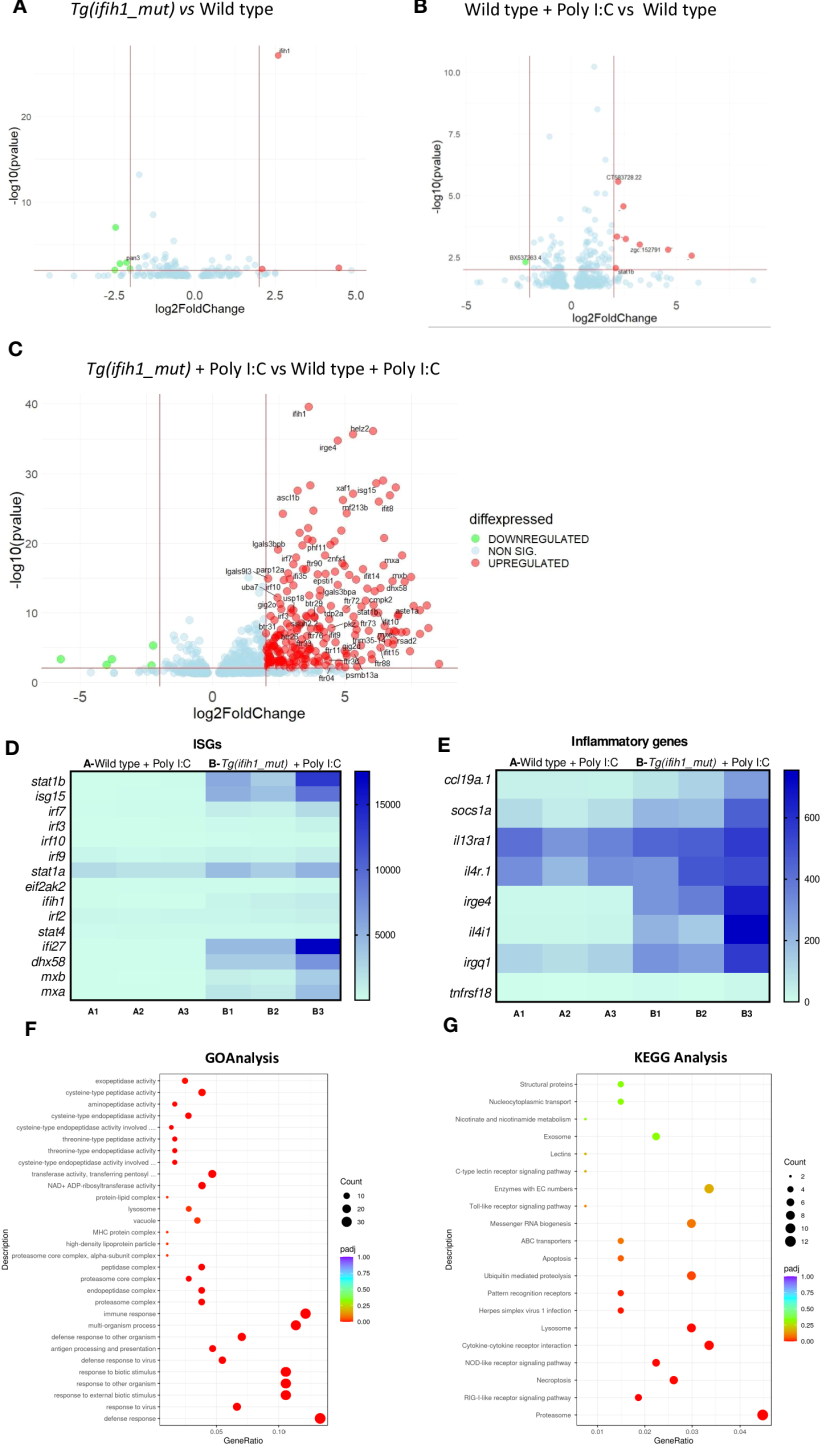
Transcriptomic analysis of the *Tg(ifih1_mut)* reveals systemic inflammation and an ISG signature upon poly I:C induction. Differentially expressed genes (DEGs) between wild-type larvae and *Tg(ifih1_mut)* **(A)**, between wild type + Poly I:C and wild-type **(B)**, and between *Tg(ifih1_mut)* + Poly I:C and wild type + Poly I:C **(C)**. **(D, E)** Heatmap of analyzed ISGs **(D)** and proinflammatory genes **(E)** between Tg(*ifih1*_mut) + Poly I:C and wild type + Poly I:C represented by counts. **(F, G)** GO **(F)** and KEGG **(G)** analysis between *Tg(ifih1_mut)* + Poly I:C and wild type + Poly I:C.

Transcriptomic analysis of 3-dpf larvae of non-injected wild-type larvae vs. Tg(*ifih1*_mut) showed similar gene expression profiles (**Figure 2A**) as well as wild type injected with a suboptimal dose of poly I:C vs. non-injected wild-type larvae (**Figure 2B**), as expected. However, when comparing wild type vs. Tg(*ifih1*_mut), both injected with the same suboptimal dose of poly I:C (**Figure 2C**), we observed a total of 872 differentially expressed genes (DEGs) (609 upregulated and 263 downregulated). From the upregulated and annotated genes, we generated a heatmap choosing several ISGs to visualize the IFN signature and found that all of them were upregulated in Tg(*ifih1*_mut) compared with wild type (**Figure 2D**), priming among them the expression of *isg15*, *stat1b*, *ifi27*, *irf7*, *mxa*, *mxb*, and *dhx58*. Other interesting upregulated genes were *helz2*, which is a helicase induced by IFN, Poly I:C, and some RNA viruses (25–27), and *irge4*, which is predicted to be involved in defense response to other organisms (28). In addition, we analyzed the transcript levels of genes encoding some major inflammatory molecules, such as *il13*, *il4*, *socs1a*, or *ccl19*, among others, that also were upregulated in Tg(*ifih1*_mut) injected with poly I:C (**Figure 2E**). Surprisingly, *tnfr18* showed similar transcript levels in poly I:C-injected Tg(*ifih1*_mut) and with wild type.

GO analysis revealed that the DEGs of *Tg(ifih1_mut)* injected with poly I:C were mainly associated with defense response to virus, immune effector processes, multi-organism process, and endopeptidase activity, among many others (**Figure 2F**). This is not surprising, as *ifih1* encodes the dsRNA receptor MDA5, which is involved in the signaling cascade activation after the recognition of viral genome. Moreover, KEGG pathway analysis demonstrated that DEGs of *Tg(ifih1_mut)* injected with poly I:C were relevant to the proteasome, RIG-like receptor signaling pathway, lysosome, necroptosis, herpes simplex virus 1 infection, and cytokine–cytokine receptor, interaction among others (**Figure 2G**). Taken together, these results confirm that the phenotype observed in *Tg(ifih1_mut)* injected with poly I:C reassembles the type I interferonopathy phenotype, which is characterized by the IFN and inflammatory signatures. In agreement with the previous study of this mutation in mice (18), we were not able to find any brain calcification or less bone mineralization (**Figure S2**) in adult transgenic zebrafish.

### Inflammation and type I IFN induction can be visualized in real time in Tg(ifih1_mut) upon poly I:C stimulation

**Figure 3:**
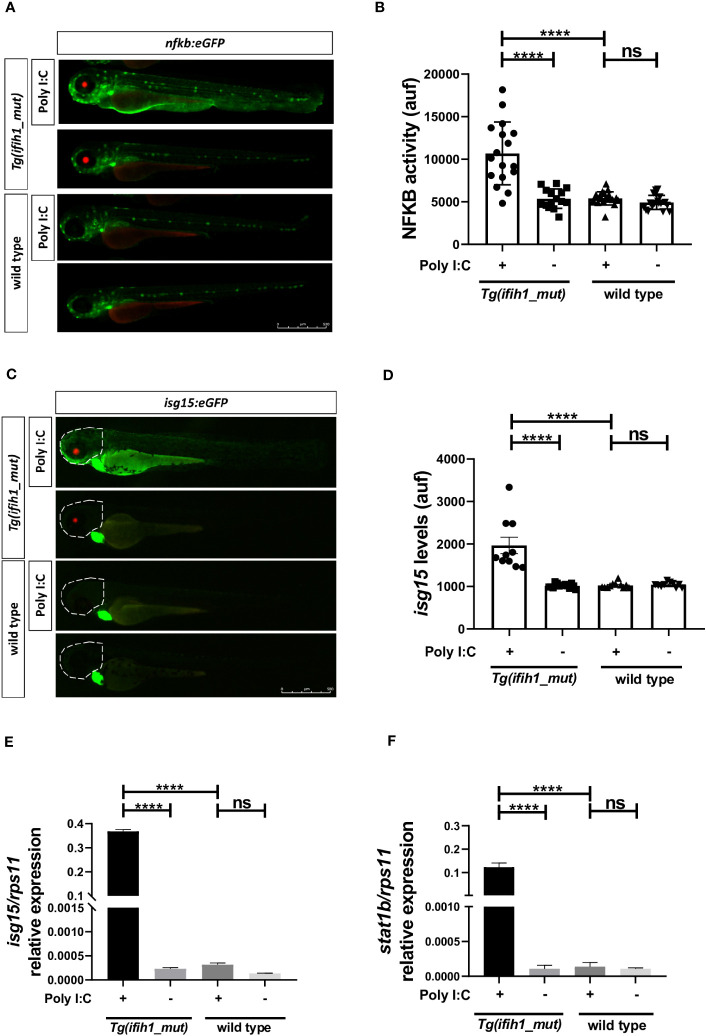
Real-time visualization of inflammation and type I IFN induction in *Tg(ifih1_mut)* upon poly I:C induction. *nfkb:eGFP* and *isg15:eGFP* (green heart marker) reporter zebrafish lines were crossed with *Tg(ifih1_mut)* (red eye marker) and injected or not with poly I:C. Nfkb activity **(A, B)** and *isg15* levels **(C, D)** were analyzed by fluorescence microscopy and quantified. Each dot represents a larva and the mean ± SEM for each experimental group is also shown. Representative merge images of whole larvae were also shown **(A, C)**. **(E, F)** Transcript levels of *isg15* and *stat1b* in larvae from the cross of *nfkb:eGFP* and *Tg(ifih1_mut)* were analyzed by RT-qPCR and the results are shown as the mean ± SEM of pooled larvae. The region of interest (ROI) used to quantify the fluorescence in *isg15:eGFP* reporter line is indicated as a dot line in the images **(C)**. *p*-values were calculated using one-way ANOVA and Tukey multiple range test. ns, not significant; ****p ≤ 0.0001.

One of the goals of generating a transgenic line carrying the *ifih1* mutation p.Arg742His was its use to perform HTS. To achieve this, we used two reporter zebrafish lines, the *nfkb:eGFP* and the *isg15:eGFP*, as an inflammation and type I IFN reporter lines, respectively. **Figures 3A**, **B**, show the transcriptional activity of Nfkb as the increase of the GFP fluorescence when crossing the Tg (*ifih1*_mut) zebrafish with the *nfkb:eGFP* reporter line and injecting poly I:C. As seen in **Figures 3A**, **B**, the fluorescence is only activated in the larvae carrying the *ifih1* p.Arg742His mutation. Similarly, increased fluorescence was observed in *Tg(ifih1_mut)* zebrafish crossed with the *isg15:eGFP* reporter line only after poly I:C stimulation (**Figures 3C, D**). As the fluorescence in this reporter line was more robust in the head area of the larvae, the quantification was made taking into account the region of interest (ROI) indicated by the dot line (**Figure 3C**). RT-qPCR expression analysis showed robustly increased transcript levels of *isg15* and *stat1b* only in larvae carrying the *ifih1* mutation p.Arg742His after poly I:C stimulation (**Figures 3E, F**), confirming the transcriptomic analysis and the results of the isg15 reporter line. All these results point out the use of the inflammation (*nfkb:eGFP*) and type I IFN (*isg15:eGFP*) reporter zebrafish lines carrying the p.Arg742His mutation to perform HTS to identify new treatments for these pathologies.

### Genetic inhibition of Mavs/Ikbke signaling pathway impairs the induction of inflammation and ISGs in Tg(ifih1_mut) upon poly I:C stimulation

**Figure 4:**
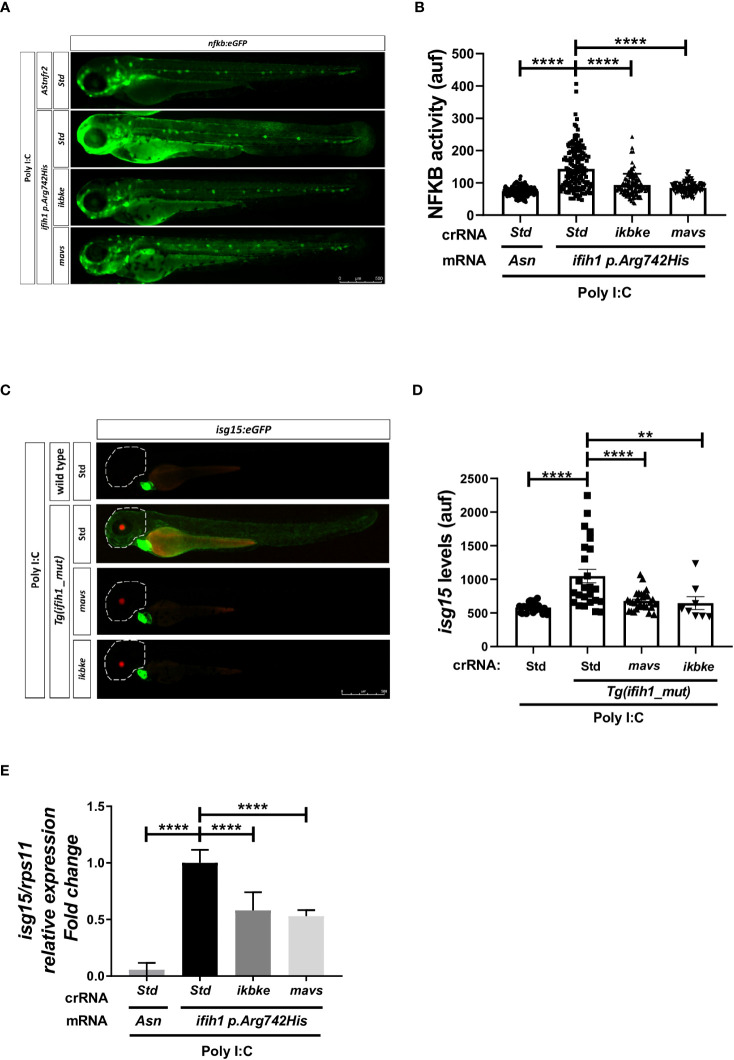
Genetic inhibition of Mavs/Ikbke signaling pathway impairs the induction of inflammation and ISGs in *Tg(ifih1_mut)* upon poly I:C stimulation. *Tg(ifih1_mut)* were crossed with *nfkb:eGFP* and *isg15:eGFP* reporter lines, injected with poly I:C and crRNA/Cas9 complexes for *mavs* or *ikbke*. Nfkb activity **(A, B)** and *isg15* levels **(C, D)** were analyzed by fluorescence microscopy and quantified. Each dot represents a larva and the mean ± SEM for each experimental group is also shown. Representative merge images of whole larvae were also shown **(A, C)**. **(E)** Transcript levels of *isg15* in larvae from the cross of *nfkb:eGFP* and *Tg(ifih1_mut)* were analyzed by RT-qPCR and the results are shown as the mean ± SEM of pooled larvae. *p*-values were calculated using one-way ANOVA and Tukey multiple range test. **p ≤ 0.01, ****p ≤ 0.0001.

To study the signal transduction through the Mda5–Mavs and Mda5–Ikbke axis and their contribution to the IFN upregulation in the reporter lines *nfkb:eGFP* and *isg15*:*eGFP*, we knock down *mavs* and *ikbke* using the CRISPR/Cas9 technology (knockdown efficiency of 60% and 62%, respectively; **Figure S3**) in *Tg(ifih1_mut).* The results showed that inhibition of either *mavs* or *ikbke* fully abrogated the induction of the fluorescence in both reporter lines (**Figures 4A–D**). Notably, mavs and ikbke deficiencies resulted in partial inhibition of *isg15* transcript levels, assayed by RT-qPCR (**Figure 4E**), indicating that the reporter lines might be more accurate to be used in HTS than RT-qPCR.

### Pharmacological inhibition of Jak impairs the induction of ISGs and inflammation in Tg(ifih1_mut) upon poly I:C stimulation

**Figure 5:**
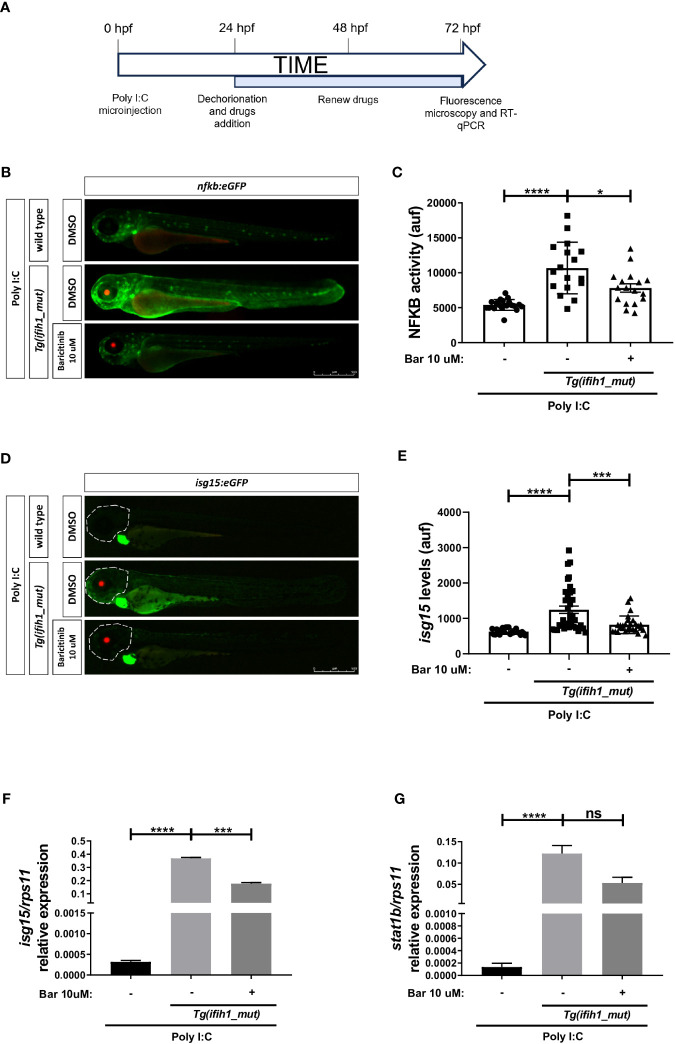
Pharmacological inhibition of Jak impairs the induction of ISGs and inflammation in *Tg(ifih1_mut)* upon poly I:C stimulation. **(A)** *nfkb:eGFP* and *isg15:eGFP* zebrafish were crossed with *Tg(ifih1_mut)*, eggs were injected with poly I:C at 0 dpf, larvae were dechorionated at 1 dpf, and baricitinib was added to the water and renewed at 2 dpf, and images were taken and analyzed by microscopy at 3 dpf. **(B–E)** Nfkb activity **(B, C)** and *isg15* levels **(D, E)** were analyzed by fluorescence microscopy and quantified. Each dot represents a larva and the mean ± SEM for each experimental group is also shown. Representative merge images of whole larvae were also shown **(B, D)**. **(F, G)** Transcript levels of *isg15* **(F)** and *stat1* **(G)** in larvae from the cross of *nfkb:eGFP* and *Tg(ifih1_mut)* were analyzed by RT-qPCR and the results are shown as the mean ± SEM of pooled larvae. *p*-values were calculated using one-way ANOVA and Tukey multiple range test. ns, not significant; *p ≤ 0.05, **p ≤ 0.01, ***p ≤ 0.001, ****p ≤ 0.0001.

To study the potential of the *Tg(ifih1_mut)* zebrafish line to be used in HTS, we performed an experiment using the commercially available JAK inhibitor baricitinib following the flow diagram shown in **Figure 5A**, and studied their ability to reduce the inflammatory and type I IFN responses using the reporter *nfkb:eGFP* and *isg15*:*eGFP*. The results showed a robust reduction of Nfkb activity (**Figures 5B, C**) and of *isg15* levels (**Figures 5D, E**) by baricitinib. These results were further confirmed by RT-PCR, where the transcript levels of *isg15* and *stat1b* showed a reduction after baricitinib treatment (**Figure 5F, G**). Taken together, these data show that pharmacological inhibition of Jak is able to reduce the inflammatory and type I IFN responses in *Tg(ifih1_mut)*, offering us the opportunity to use them in HTS that will hopefully find new treatments for interferonopathies.

## Discussion

Type I interferonopathies have been widely studied in the past decades, and there are still many questions to be answered to properly understand these pathologies and propose new treatments that are still scarce for these patients. Inside the wide spectrum of type I interferonopathies, we can find AGS, SMS, and SLE, which have different clinical manifestations, but that coincide in a common characteristic, an upregulated systemic type I IFN expression. Among the many genes responsible for the appearance of type I interferonopathies, there are two, RIG-I and MDA5, that can cause SMS or AGS depending on the mutated amino acid, demonstrating the importance in the selection of the mutation for the study. In this work, we focused on the human mutation of *IFIH1* p.Arg779His, a mutation that causes AGS and monogenic lupus and it has also been found to cause systemic type I IFN upregulation in a mouse model (18). This mutation is located in the Hel2 domain of *IFIH1* whose sequence is highly conserved between species, including zebrafish (p.Arg742His).

Transgenic zebrafish ubiquitously expressing the *ifih1* p.Arg742His mutation showed systemic inflammation and IFN signature, as found in patients, after the induction with suboptimal doses of poly I:C. Strikingly, this line developed normally, showed normal life span, and poly I:C stimulation is required to induce inflammation and type I IFN in larvae, contrasting the mouse model R779H that spontaneously develops myocarditis and nephritis due to overproduction of type I IFN in major organs (18). As for most of the mouse models described so far, our zebrafish model presents the same limitation regarding the study of the neurological pathologies, as we do not detect any neurological phenotype in our zebrafish model, such as any anomaly in their movement or different head morphology as microcephaly. It has been widely studied that the appearance of pathological alterations in several organs of mouse models of IFIH1-mediated interferonopathy is directly related to the overproduction of type I IFN, as the depletion of the IFNAR (interferon-α/β receptor) or adaptor molecules in the signaling route, such as MAVS, abrogates these alterations (18–20, 29). Moreover, the use of JAK inhibitors to abrogate the IFN signal in patients also improve the clinical symptoms in patients suffering from juvenile dermatomyositis, Still´s disease, CANDLE, SAVI, and other interferonopathies (30–32), suggesting that the discovery of new compounds that limit the expression of IFN would be of direct interest for the prevention of the global phenotype, including neurological alterations. Therefore, it is not surprising that the *Tg(ifih1_mut)* zebrafish model does not present any alteration as it does not have a constitutive activation of type I IFN. It remains to be investigated why the *Tg(ifih1_mut)* zebrafish line does not show baseline IFN signaling, while conserving increased sensitivity to ligand-induced IFN signaling.

The transcriptomic analysis of *Tg(ifih1_mut)* after poly I:C induction revealed that they develop type I IFN production and inflammatory responses compared with their wild-type siblings after poly I:C induction, which was manifested by the upregulation of ISGs and proinflammatory genes, indicating that the mutation conferred a higher sensitivity to dsRNA recognition. The gene expression profiles of *Tg(ifih1_mut)* were similar to the ones of the *Ifih1* mutant mouse model (18) and human patients (2, 24). Moreover, GO analysis of DEGs and KEGGs showed that the DEGs were related to response to virus, immune effector processes or RLR signaling pathways, which is logical, as Mda5 is directly involved in the activation of the signaling cascade after exogenous viral genome recognition. Therefore, the phenotype observed in *Tg(ifih1_mut)* after poly I:C injection recapitulates the IFN signature and inflammatory response seen in human interferonopathy patients.

One of the most important results of this study is that *Tg(ifih1_mut)* is amenable for *in vivo* imaging and HTS. Thus, combining *Tg(ifih1_mut)* with the type I IFN and inflammation reporter lines *isg15*:*eGFP* and *nfkb:eGFP*, respectively, allowed us to visualize in real time and in a whole vertebrate organism the induction of ISGs and the inflammatory response, avoiding time-consuming and expensive RT-qPCR. Unexpectedly, both reported lines seemed to be more accurate than RT-qPCR to quantitate ISG induction and inflammation, i.e., Nfkb activity, in *Tg(ifih1_mut)* upon poly I:C stimulation.

**Figure 6:**
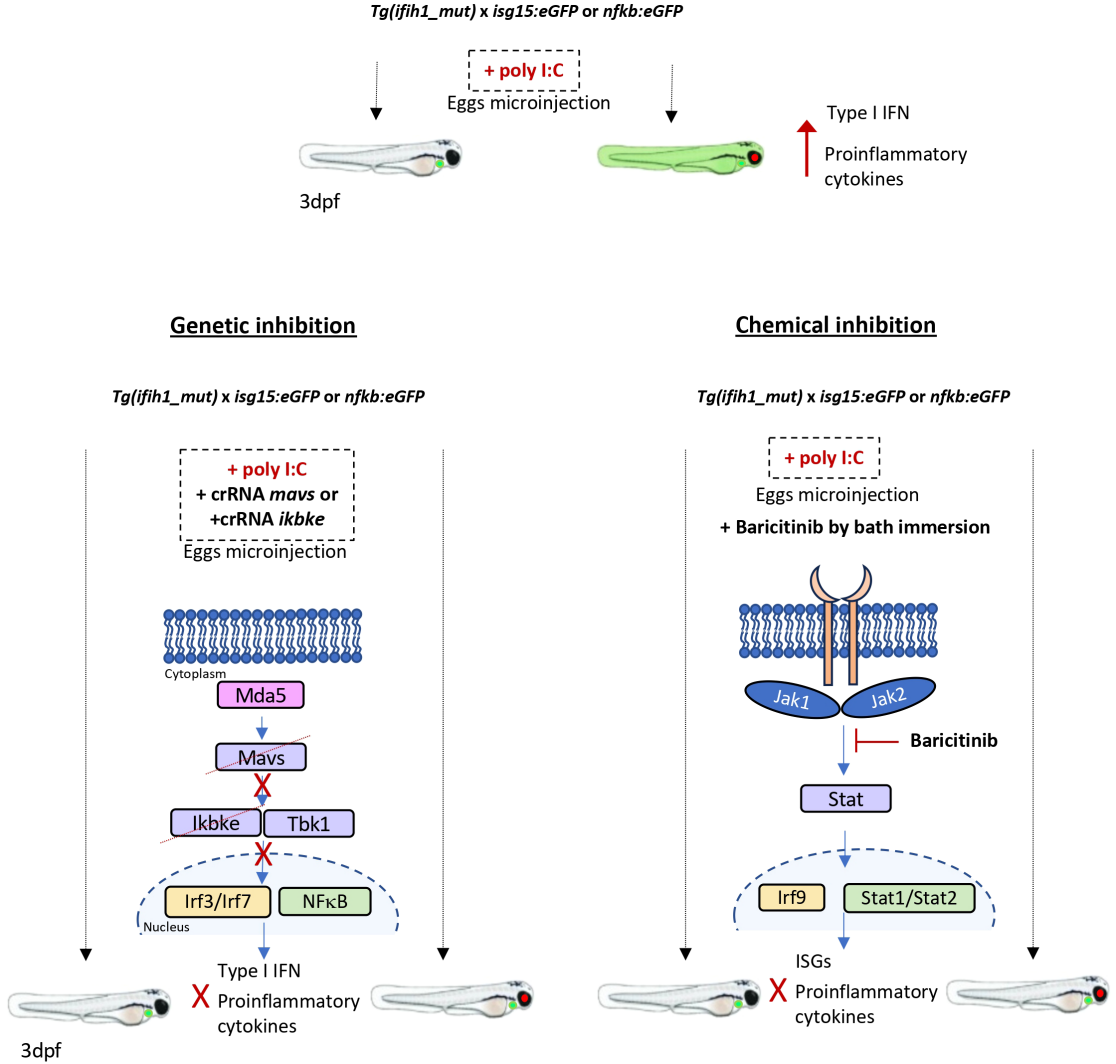
Schematic summary. *Tg(ifih1_mut)* crossed with the inflammation and type I interferon reporters, *nfkb:eGFP* and *isg15:eGFP*, activate the expression of GFP 3 days after the injection of poly I:C. Larvae bearing the mutation p.Arg742His show the red eye tag, and larvae bearing the *isg15:eGFP* reporter show the green heart tag marker. The genetic inhibition of *mavs* or *ikbke* prevents the transduction of the signal from Mda5 after sensing the poly I:C, avoiding the activation and translocation of Irf3/irf7 and Nfkb into the nucleus and preventing the expression of type I interferon and proinflammatory cytokines and the GFP activation. The chemical inhibition by the addition of Baricitinib by bath immersion provokes the blockade of the signal transduction through the Jak1 and Jak2 kinases, which results in the prevention of Stat and Irf9 translocation into the nucleus and activation of the ISGs and proinflammatory cytokine expression, avoiding the activation of GFP of the reporter lines.

Genetic and pharmacological inhibition experiments showed that both inflammation and type I IFN production in *Tg(ifih1_mut)* depended on Mavs/Ikbke and Jak signaling pathways, as occurs in the mouse model of IFIH1-driven interferonopathy and human patients (18–20, 33). In fact, JAK inhibitors are the usual treatment for patients suffering from type I interferonopathies (30, 33, 34). Although JAK inhibitors are able to reverse the IFN signature, the inflammatory response, and the pathological alteration observed in mouse models of type I interferonopathies caused by *IFIH1* mutations, and in other genes (18–20), it has been pointed out that the use of mice to test drugs is technically difficult, as the therapy has to start at early life stages, where the administration can be very laborious (18). In zebrafish, however, we have demonstrated that the administration of JAK inhibitors by bath is sufficient to abrogate the activation of the type I IFN and the inflammatory response (**Figure 6**) and this can be achieved in real time with minimal manipulation using the reporter lines *isg15*:*eGFP* and *nfkb*:*eGFP*. Interestingly, JAK inhibitors were able to reduce not only the type I IFN activation, but also the inflammatory response, indicating that their use can be also beneficial to reduce the negative effects of exacerbated inflammation associated to type I interferonopathies. Therefore, the zebrafish model of type I interferonopathies reported in this study is an excellent tool for the identification of novel or the repurposing of FDA/EMA-approved drugs to treat these devastating diseases.

## Materials and methods

### Animals

Zebrafish (*Danio rerio* H.) were obtained from the Zebrafish International Resource Center and mated, staged, raised, and processed as described (35). The lines *Tg(6xNFKB:eGFP)^sh235^* referred to as a *nfkb:eGFP* (36), Tg(*isg15:GFP*) (37) were previously described. The transgenic line Tg(*bactin2:ifih1p.Arg742His;cryaa:RFP*), referred to as *Tg(ifih1_mut)*, was constructed using the Tol2 transposon construct bearing the promoter from *actb2* fused to the coding sequence of *ifih1* bearing the mutation together with a CFP driven by the eye-specific promoter *creaa* in the opposite orientation. All the experiments complied with the Guidelines of the European Union Council (Directive 2010/63/EU) and Spanish Royal Decree, RD 53/2013. Experiments and procedures were performed as approved by the Bioethical Committees of the University of Murcia (CEEA 669/2020).

### Analysis of gene expression

Total RNA was extracted from a pool of 25 zebrafish larvae (3 dpf) with TRIzol reagent (Invitrogen) following the manufacturer’s instructions and treated with DNase I, amplification grade (1 U/μg RNA, Invitrogen). SuperScript VILO cDNA Synthesis Kit (Invitrogen) was used to synthesize first-strand cDNA with random primer from 1 μg of total RNA at 50°C for 50 min. Real-time PCR was performed with an ABIPRISM 7500 instrument (Applied Biosystems) using SYBR Green PCR Core Reagents (Applied Biosystems). Reaction mixtures were incubated for 10 min at 95°C, followed by 40 cycles of 15 s at 95°C, 1 min at 60°C, and finally 15 s at 95°C, 1 min 60°C, and 15 s at 95°C. For each mRNA, gene expression was normalized to the ribosomal protein S11 gene (*rps11*) content in each sample using the Pfaffl method (27). The primer sequences used are listed in **Supplementary Table 1**. In all cases, each PCR was performed with triplicate samples and repeated at least twice with independent samples. A representative experiment out of three independent biological replicates is shown in figures.

### *In vivo* imaging

Nfkb activity and *isg15* levels were determined using *Tg(nfkb:eGFP)* and *Tg(isg15:GFP)* lines, respectively. Briefly, 3-dpf zebrafish larvae were anesthetized in embryo medium with 0.16 mg/mL buffered tricaine. Images of complete larvae were taken at 3 dpf using a Leica MZ16FA fluorescence stereomicroscope. The fluorescence intensity was obtained and analyzed with ImageJ (FIJI) software from three biological replicates each containing several larvae.

### TAC imaging

Three wild-type and three *Tg(ifih1_mut)* zebrafish (1 year old) were used for analysis of the bone mineralization. The fish were sacrificed by treating them with tricaine 2× (0.32 mg/mL) and then subjected to microcomputed tomography (mCT) and histomorphometric analyses, respectively. The scanning was performed using a Quantum GX2 microCT scanner, with the following parameters; Kv: 90, μA: 80, FOV: 10 mm, voxel size: 20 μm, scan mode: high resolution, scan time: 4 min, absorbed dose: 842 mGy, X-ray filter: Cu 0.06+Al 0.5. Three-dimensional microstructural image data were reconstructed and analyzed using OsiriX MD 13.0.2 software (Pixmeo, Bernex, Switzerland). Bone densities were measured in ROIs of the head with a minimum and maximum threshold of 500 and 6,500, respectively. These densities were determined by Hounsfield units (HU) and compared in wild-type and *Tg(ifih1_mut)* zebrafish.

### RNA-sequencing

Four groups of 3-dpf larvae from crossing *nfkb:eGFP* and *Tg(ifih1_mut)*, namely, the unstimulated wild type, wild type stimulated with poly (I:C), unstimulated, and stimulated with poly (I:C) Tg(*ifih1*_mut), were selected for RNA Seq analysis of DEGs. In each group, 25 larvae were pooled, washed, and frozen in liquid nitrogen. RNA isolation, RNA quality evaluation (yield, purity, and integrity), cDNA library construction, and Illumina sequencing were performed by NovoGene (38). The DEGs between the two samples were identified if they had a log2Foldchange < −0.5 or > 0.5 and *q* (adjusted *p*-value) < 0.05 (39). A Hierarchical clustering analysis of DEGs was performed. Gene ontology and KEGG pathway analyses were performed to analyze the gene enrichment of the DEGs, and those with corrected *p* < 0.05 and *p* < 0.05 were considered as significantly enriched Gene ontology terms and pathways, respectively. Raw data together with the original data tables have been included in the GEO Repository with the reference GSE244292.

### CRISPR/Cas9 and mRNA injections and chemical treatments in zebrafish

CRISPR RNA (crRNA) for zebrafish *mavs* and *ikbke* (**Table S2**) and negative control crRNA (catalog no. 1072544, crSTD) and tracrRNA (trans-activating tracrRNA; catalog no. 1072533) were purchased from Integrated DNA Technologies (IDT) and resuspended in nuclease-free duplex buffer to 100 µM. One microliter of each was mixed and incubated for 5 min at 95°C for duplexing. After removing from the heat and cooling to room temperature, 1.43 µL of nuclease-free duplex buffer was added to the duplex (gRNA, crRNA + tracrRNA), giving a final concentration of 1,000 ng/µL. Last, the injection mix was prepared by mixing 1 µL of duplex, 2.55 µL of nuclease-free duplex buffer, 0.25 µL of Cas9 nuclease V3 (IDT, catalog no. 1081058), and 0.25 µL of phenol red, giving final concentrations of gRNA duplex (250 ng/µL) and of Cas9 (500 ng/µL). The prepared mix was microinjected into the yolk of one-cell-stage embryos using a microinjector (Narishige) (0.5 to 1 nL per embryo). The same amounts of gRNA were used in all the experimental groups. The efficiency of gRNA was checked by amplifying the target sequence with a specific pair of primers (**Table S1**) and the TIDE webtool (https://tide.nki.nl/) (**Figure S3**).

*In vitro*–transcribed RNA was obtained following the manufacturer’s instructions (mMESSAGE mMACHINE kit, Ambion). RNA was mixed in microinjection buffer and microinjected into the yolk of one-cell-stage embryos using a microinjector (Narishige; 0.5 to 1 nL per embryo). The same amount of RNA was used for all the experimental groups.

Poly (I:C) HMW (InvivoGen, #tlrl-pic) was microinjected into the yolk of one-cell-stage embryos (25 pg/embryo). One-dpf embryos were manually dechorionated and treated from 2 days by chemical bath immersion at 28°C. Incubation was carried out in six-well plates containing 25 larvae/well in egg water (including 60 μg/mL sea salts in distilled water) supplemented with 1% dimethyl sulfoxide (DMSO). Baricitinib (Bar) was added to the water at 10 µM.

### Statistical analyses

All statistical analyses were performed in GraphPad Prism 8. Data are shown as mean ± SEM and were analyzed by one-way analysis of variance (ANOVA) and a Tukey multiple range test to determine differences between groups. The differences between two samples were analyzed by Student’s *t*-test.

## Data availability statement

The original contributions presented in the study are publicly available. This data can be found here: https://www.ncbi.nlm.nih.gov/; GSE244292.

## Ethics statement

The animal study was approved by Guidelines of the European Union Council (Directive 2010/63/EU) and Spanish Royal Decree, RD 53/2013. The study was conducted in accordance with the local legislation and institutional requirements.

## Author contributions

BB-B: Investigation, Methodology, Writing – review & editing. AM-L: Conceptualization, Investigation, Writing – original draft, Methodology. FM: Investigation, Methodology, Writing – review & editing. ST: Investigation, Methodology, Writing – review & editing. TM-M: Investigation, Writing – review & editing. PM-d-C: Investigation, Writing – review & editing. MC: Investigation, Writing – review & editing. VM: Writing – review & editing, Investigation. DG-M: Conceptualization, Investigation, Writing – review & editing.

## Supplementary material

Supplementary Figure 1 Poly I:C dose curve. Wild type eggs were injected with 10, 50, 100 pg/egg of poly I:C and *isg15* expression was measured by RT-PCR at 3 dpi **(A)**. Three dpi larvae from eggs injected with Asn mRNA or *ifih1* p.Arg742His mRNA with or without 25 pg/egg of poly I:C **(B)**.

Supplementary Figure 2 Analysis of bone density in 1-year-old *Tg(ifih1_mut)*. **(A)** Bone densities from three adult *Tg(ifih1_mut)* and three wild-type zebrafish were measured in regions of interest (ROI) of the head with a minimum and maximum threshold of 500 and 6500, respectively. These densities were determined by Hounsfield units (HU). **(B)** Representative image from the bone quantified region. **(C)** Representative image from the brain TAC, transversal cut upper panels and longitudinal cut lower panels.

Supplementary Figure 3 Analysis of the efficiency of CRISPR/Cas9 edition. The efficiency of each crRNA was checked by amplifying the target sequence with a specific pair of primers (see **Table S1**) and the TIDE webtool (https://tide.nki.nl/). **(A)** *mavs* and **(B)** *ikbke* indel spectrum.

## References

[1] Schneider WM Chevillotte MD Rice CM. Interferon-stimulated genes: a complex web of host defenses. Annu Rev Immunol (2014) 32:513–45. doi: 10.1146/annurev-immunol-032713-120231 PMC4313732 24555472

[2] Rice GI Del Toro Duany Y Jenkinson EM Forte GM Anderson BH Ariaudo G. Gain-of-function mutations in IFIH1 cause a spectrum of human disease phenotypes associated with upregulated type I interferon signaling. Nat Genet (2014) 46(5):503–9. doi: 10.1038/ng.2933 PMC4004585 24686847

[3] Funabiki M Kato H Miyachi Y Toki H Motegi H Inoue M. Autoimmune disorders associated with gain of function of the intracellular sensor MDA5. Immunity (2014) 40(2):199–212. doi: 10.1016/j.immuni.2013.12.014 24530055

[4] Rutsch F MacDougall M Lu C Buers I Mamaeva O Nitschke Y. A specific IFIH1 gain-of-function mutation causes Singleton-Merten syndrome. Am J Hum Genet (2015) 96(2):275–82. doi: 10.1016/j.ajhg.2014.12.014 PMC4320263 25620204

[5] Pettersson M Bergendal B Norderyd J Nilsson D Anderlid B-M Nordgren A. Further evidence for specific IFIH1 mutation as a cause of Singleton-Merten syndrome with phenotypic heterogeneity. Am J Med Genet A (2017) 173(5):1396–9. doi: 10.1002/ajmg.a.38214 28319323

[6] Singleton EB Merten DF. An unusual syndrome of widened medullary cavities of the metacarpals and phalanges, aortic calcification and abnormal dentition. Pediatr Radiol (1973) 1(1):2–7. doi: 10.1007/BF00972817 4272099

[7] Gay BB Jr. Kuhn JP. A syndrome of widened medullary cavities of bone, aortic calcification, abnormal dentition, and muscular weakness (the Singleton-Merten syndrome). Radiology (1976) 118(2):389–95. doi: 10.1148/118.2.389 175395

[8] Valverde I Rosenthal E Tzifa A Desai P Bell A Pushparajah K. Singleton-merten syndrome and impaired cardiac function. J Am Coll Cardiol (2010) 56(21):1760. doi: 10.1016/j.jacc.2010.02.078 21070929

[9] Crow YJ Hayward BE Parmar R Robins P Leitch A Ali M. Mutations in the gene encoding the 3'-5' DNA exonuclease TREX1 cause Aicardi-Goutieres syndrome at the AGS1 locus. Nat Genet (2006) 38(8):917–20. doi: 10.1038/ng1845 16845398

[10] Crow YJ Leitch A Hayward BE Garner A Parmar R Griffith E. Mutations in genes encoding ribonuclease H2 subunits cause Aicardi-Goutieres syndrome and mimic congenital viral brain infection. Nat Genet (2006) 38(8):910–6. doi: 10.1038/ng1842 16845400

[11] Rice GI Bond J Asipu A Brunette RL Manfield IW Carr IM. Mutations involved in Aicardi-Goutieres syndrome implicate SAMHD1 as regulator of the innate immune response. Nat Genet (2009) 41(7):829–32. doi: 10.1038/ng.373 PMC4154505 19525956

[12] Rice GI Kasher PR Forte GMA Mannion NM Greenwood SM Szynkiewicz M. Mutations in ADAR1 cause Aicardi-Goutieres syndrome associated with a type I interferon signature. Nat Genet (2012) 44(11):1243–8. doi: 10.1038/ng.2414 PMC4154508 23001123

[13] Uggenti C Lepelley A Depp M Badrock AP Rodero MP El-Daher M-T. cGAS-mediated induction of type I interferon due to inborn errors of histone pre-mRNA processing. Nat Genet (2020) 52(12):1364–72. doi: 10.1038/s41588-020-00737-3 33230297

[14] Naesens L Nemegee J Roelens F Vallaeys L Meuwissen M Janssens K. Mutations in RNU7-1 weaken secondary RNA structure, induce MCP-1 and CXCL10 in CSF, and result in aicardi-goutieres syndrome with severe end-organ involvement. J Clin Immunol (2022) 42(5):962–74. doi: 10.1007/s10875-022-01209-5 PMC9402729 35320431

[15] Roers A Hiller B Hornung V. Recognition of endogenous nucleic acids by the innate immune system. Immunity (2016) 44(4):739–54. doi: 10.1016/j.immuni.2016.04.002 27096317

[16] Bursztejn AC Briggs TA del Toro Duany Y Anderson BH O'Sullivan J Williams SG. Unusual cutaneous features associated with a heterozygous gain-of-function mutation in IFIH1: overlap between Aicardi-Goutieres and Singleton-Merten syndromes. Br J Dermatol (2015) 173(6):1505–13. doi: 10.1111/bjd.14073 PMC4745891 26284909

[17] Gruber C Bogunovic D. Incomplete penetrance in primary immunodeficiency: a skeleton in the closet. Hum Genet (2020) 139(6-7):745–57. doi: 10.1007/s00439-020-02131-9 PMC7275875 32067110

[18] Ohto T Tayeh AA Nishikomori R Abe H Hashimoto K Baba S. Intracellular virus sensor MDA5 mutation develops autoimmune myocarditis and nephritis. J Autoimmun (2022) 127:102794. doi: 10.1016/j.jaut.2022.102794 35168003

[19] Soda N Sakai N Kato H Takami M Fujita T. Singleton-merten syndrome-like skeletal abnormalities in mice with constitutively activated MDA5. J Immunol (2019) 203(5):1356–68. doi: 10.4049/jimmunol.1900354 31366715

[20] Emralino FL Satoh S Sakai N Takami M Takeuchi F Yan N. Double-stranded RNA induces mortality in an MDA5-mediated type I interferonopathy model. J Immunol (2022) 209(11):2093–103. doi: 10.4049/jimmunol.2200367 36426976

[21] Oda H Nakagawa K Abe J Awaya T Funabiki M Hijikata A. Aicardi-Goutieres syndrome is caused by IFIH1 mutations. Am J Hum Genet (2014) 95(1):121–5. doi: 10.1016/j.ajhg.2014.06.007 PMC4085581 24995871

[22] Rice GI Park S Gavazzi F Adang LA Ayuk LA Van Eyck L. Genetic and phenotypic spectrum associated with IFIH1 gain-of-function. Hum Mutat (2020) 41(4):837–49. doi: 10.1002/humu.23975 PMC7457149 31898846

[23] Ruaud L Rice GI Cabrol C Piard J Rodero M Van Eyck L. Autosomal-dominant early-onset spastic paraparesis with brain calcification due to IFIH1 gain-of-function. Hum Mutat (2018) 39(8):1076–80. doi: 10.1002/humu.23554 PMC6043383 29782060

[24] de Carvalho LM Ngoumou G Park JW Ehmke N Deigendesch N Kitabayashi N. Musculoskeletal disease in MDA5-related type I interferonopathy: A mendelian mimic of jaccoud's arthropathy. Arthritis Rheumatol (2017) 69(10):2081–91. doi: 10.1002/art.40179 PMC6099183 28605144

[25] Schoggins JW Wilson SJ Panis M Murphy MY Jones CT Bieniasz P. A diverse range of gene products are effectors of the type I interferon antiviral response. Nature (2011) 472(7344):481–5. doi: 10.1038/nature09907 PMC3409588 21478870

[26] Fusco DN Brisac C John SP Huang Y-W Chin CR Xie T. A genetic screen identifies interferon-alpha effector genes required to suppress hepatitis C virus replication. Gastroenterology (2013) 144(7):1438–49, 1449 e1-9. doi: 10.1053/j.gastro.2013.02.026 23462180 PMC3665646

[27] Xie X Liu PS Percipalle P. Analysis of Global Transcriptome Change in Mouse Embryonic Fibroblasts After dsDNA and dsRNA Viral Mimic Stimulation. Front Immunol (2019) 10:836. doi: 10.3389/fimmu.2019.00836 31057555 PMC6478819

[28] Sieger D Stein C Neifer D van der Sar AM Leptin M. The role of gamma interferon in innate immunity in the zebrafish embryo. Dis Model Mech (2009) 2(11-12):571–81. doi: 10.1242/dmm.003509 19779068

[29] Crampton SP Deane JA Feigenbaum L Bolland S. Ifih1 gene dose effect reveals MDA5-mediated chronic type I IFN gene signature, viral resistance, and accelerated autoimmunity. J Immunol (2012) 188(3):1451–9. doi: 10.4049/jimmunol.1102705 PMC3262963 22205024

[30] Sanchez GAM Reinhardt A Ramsey S Wittkowski H Hashkes PJ Berkun Y. JAK1/2 inhibition with baricitinib in the treatment of autoinflammatory interferonopathies. J Clin Invest (2018) 128(7):3041–52. doi: 10.1172/JCI98814 PMC6026004 29649002

[31] Le Voyer T Gitiaux C Authier F-J Bodemer C Melki I Quartier P. JAK inhibitors are effective in a subset of patients with juvenile dermatomyositis: a monocentric retrospective study. Rheumatol (Oxford) (2021) 60(12):5801–8. doi: 10.1093/rheumatology/keab116 33576769

[32] Gillard L Pouchot J Cohen-Aubart F Koné-Paut I Mouterde G Michaud M. JAK inhibitors in difficult-to-treat adult-onset Still's disease and systemic-onset juvenile idiopathic arthritis. Rheumatol (Oxford) (2023) 62(4):1594–604. doi: 10.1093/rheumatology/keac440 35920788

[33] Kothur K Bandodkar S Chu S Wienholt L Johnson A Barclay P. An open-label trial of JAK 1/2 blockade in progressive IFIH1-associated neuroinflammation. Neurology (2018) 90(6):289–91. doi: 10.1212/WNL.0000000000004921 29321238

[34] Vanderver A Adang L Gavazzi F McDonald K Helman G Frank DB. Janus kinase inhibition in the aicardi-goutieres syndrome. N Engl J Med (2020) 383(10):986–9. doi: 10.1056/NEJMc2001362 PMC7495410 32877590

[35] Westerfield M. A guide for the laboratory use of zebrafish (Danio rerio) Eugene Vol. 1. Institute of Neuroscience, University of Oregon, Eugene: University of Oregon Press (2020) p. 10–6.

[36] Kanther M Sun X Mühlbauer M Mackey LC Flynn EJ 3rd Bagnat M. Microbial colonization induces dynamic temporal and spatial patterns of NF-kappaB activation in the zebrafish digestive tract. Gastroenterology (2011) 141(1):197–207. doi: 10.1053/j.gastro.2011.03.042 21439961 PMC3164861

[37] Balla KM Rice MC Gagnon JA Elde NC. Linking virus discovery to immune responses visualized during zebrafish infections. Curr Biol (2020) 30(11):2092–2103 e5. doi: 10.1016/j.cub.2020.04.031 32413307 PMC7854381

[38] Zhang X Zhou Q Zou W Hu X. Molecular mechanisms of developmental toxicity induced by graphene oxide at predicted environmental concentrations. Environ Sci Technol (2017) 51(14):7861–71. doi: 10.1021/acs.est.7b01922 28614664

[39] Thakur M Crow M Richards N Davey GIJ Levine E Kelleher JH. Defining the nociceptor transcriptome. Front Mol Neurosci (2014) 7:87. doi: 10.3389/fnmol.2014.00087 25426020 PMC4227287

