# Corrigendum: A zebrafish model of Ifih1-driven Aicardi–Goutières syndrome reproduces the interferon signature and the exacerbated inflammation of patients

**DOI:** 10.3389/fimmu.2024.1544703

**Published:** 2025-01-06

**Authors:** Beatriz Bernal-Bermúdez, Alicia Martínez-López, Francisco J. Martínez-Morcillo, Sylwia D. Tyrkalska, Teresa Martínez-Menchón, Pablo Mesa-del-Castillo, María L. Cayuela, Victoriano Mulero, Diana García-Moreno

**Affiliations:** ^1^ Departamento de Biología Celular e Histología, Facultad de Biología, Universidad de Murcia, Murcia, Spain; ^2^ Instituto Murciano de Investigación Biosanitaria (IMIB)-Pascual Parrilla, Murcia, Spain; ^3^ Centro de Investigación Biomédica en Red de Enfermedades Raras (CIBERER), Instituto de Salud Carlos III, Madrid, Spain; ^4^ Hospital Clínico Universitario Virgen de la Arrixaca, Murcia, Spain

**Keywords:** type I IFN, IFIH1, zebrafish avatar, autoimmunity, drug screening

In the published article, there was an error in the Funding statement. The research grant which appeared in the published **Funding** statement was incorrect. The correct **Funding** statement appears below.

“The author(s) declare financial support was received for the research, authorship, and/or publication of this article. This study has been funded by Instituto de Salud Carlos III (ISCIII) through the project “CP21/00028” and research grant PI22/00879 and co-funded by the European Union to DG-M) and Consejería de Salud de la Región de Murcia (ZEBER project to MC and VM), Spanish Ministry of Science and Innovation (Juan de la Cierva-Incorporacion postdoctoral contract to ST). The funders had no role in the study design, data collection and analysis, decision to publish, or preparation of the manuscript.”

The authors apologize for this error and state that this does not change the scientific conclusions of the article in any way. The original article has been updated.

