# Supplementary figures and images for: A zebrafish model of Ifih1-driven Aicardi–Goutières syndrome reproduces the interferon signature and the exacerbated inflammation of patients
Source: Front Immunol. 2023 Nov 24;14:1294766. doi: 10.3389/fimmu.2023.1294766 (PMC10704509; doi:10.3389/fimmu.2023.1294766)

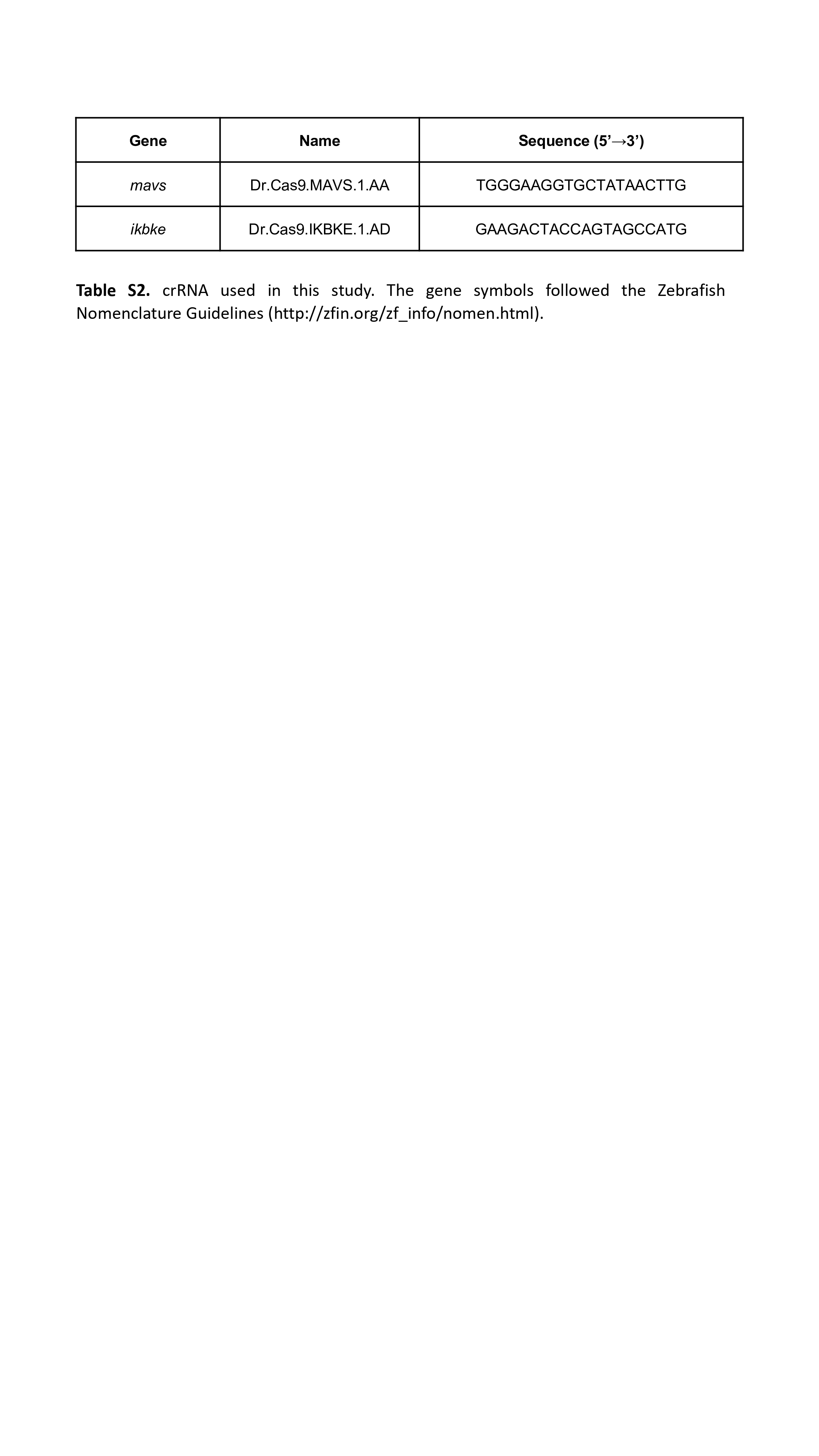

Supplement: Supplementary Figure 1 — Poly I:C dose curve. Wild type eggs were injected with 10, 50, 100 pg/egg of poly I:C and isg15 expression was measured by RT-PCR at 3 dpi (A). Three dpi larvae from eggs injected with Asn mRNA or ifih1 p.Arg742His mRNA with or without 25 pg/egg of poly I:C (B). [file Image_1.jpeg]

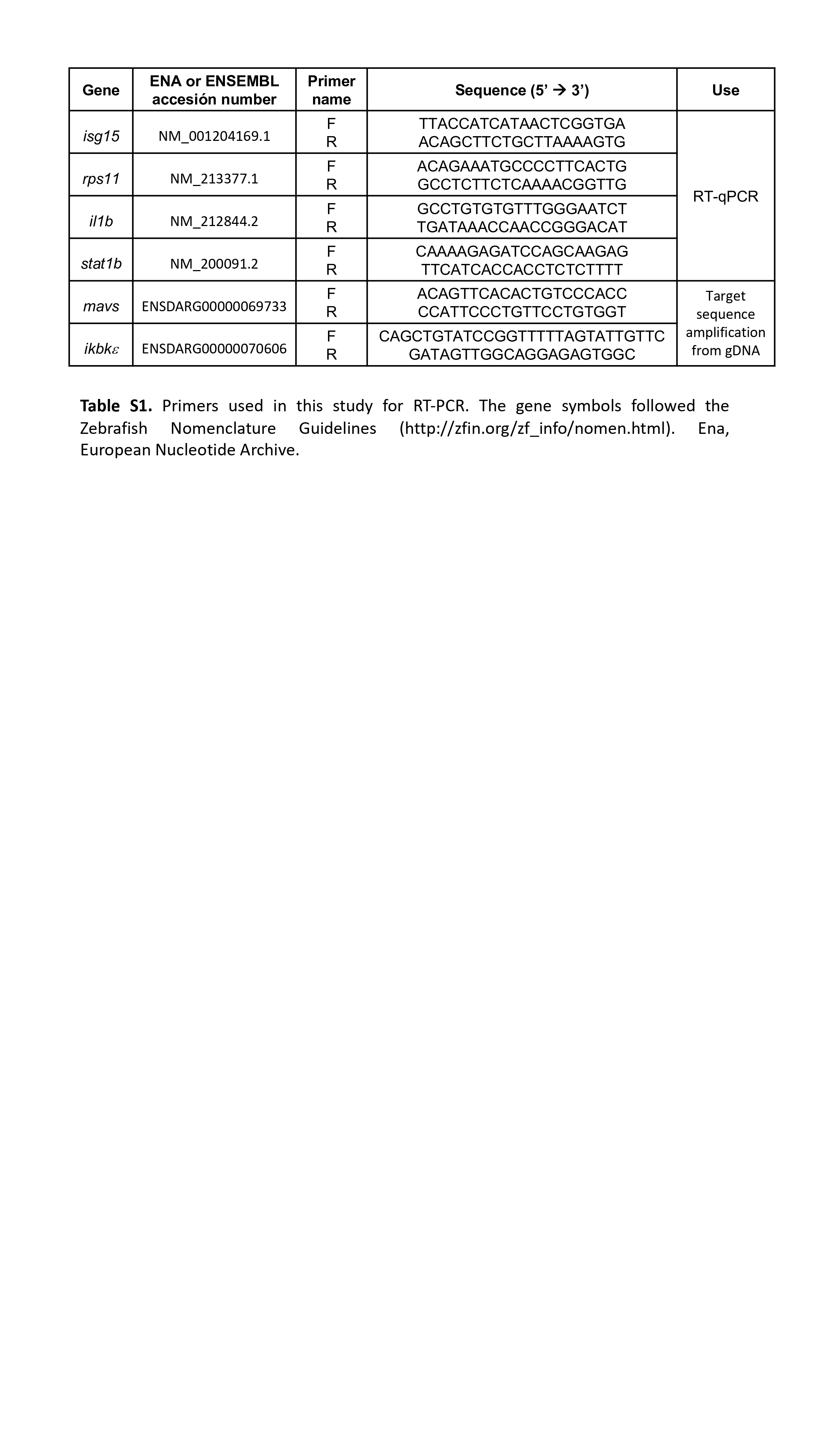

Supplement: Supplementary Figure 2 — Analysis of bone density in 1-year-old Tg(ifih1_mut). (A) Bone densities from three adult Tg(ifih1_mut) and three wild-type zebrafish were measured in regions of interest (ROI) of the head with a minimum and maximum threshold of 500 and 6500, respectively. These densities were determined by Hounsfield units (HU). (B) Representative image from the bone quantified region. (C) Representative image from the brain TAC, transversal cut upper panels and longitudinal cut lower panels. [file Image_2.jpeg]

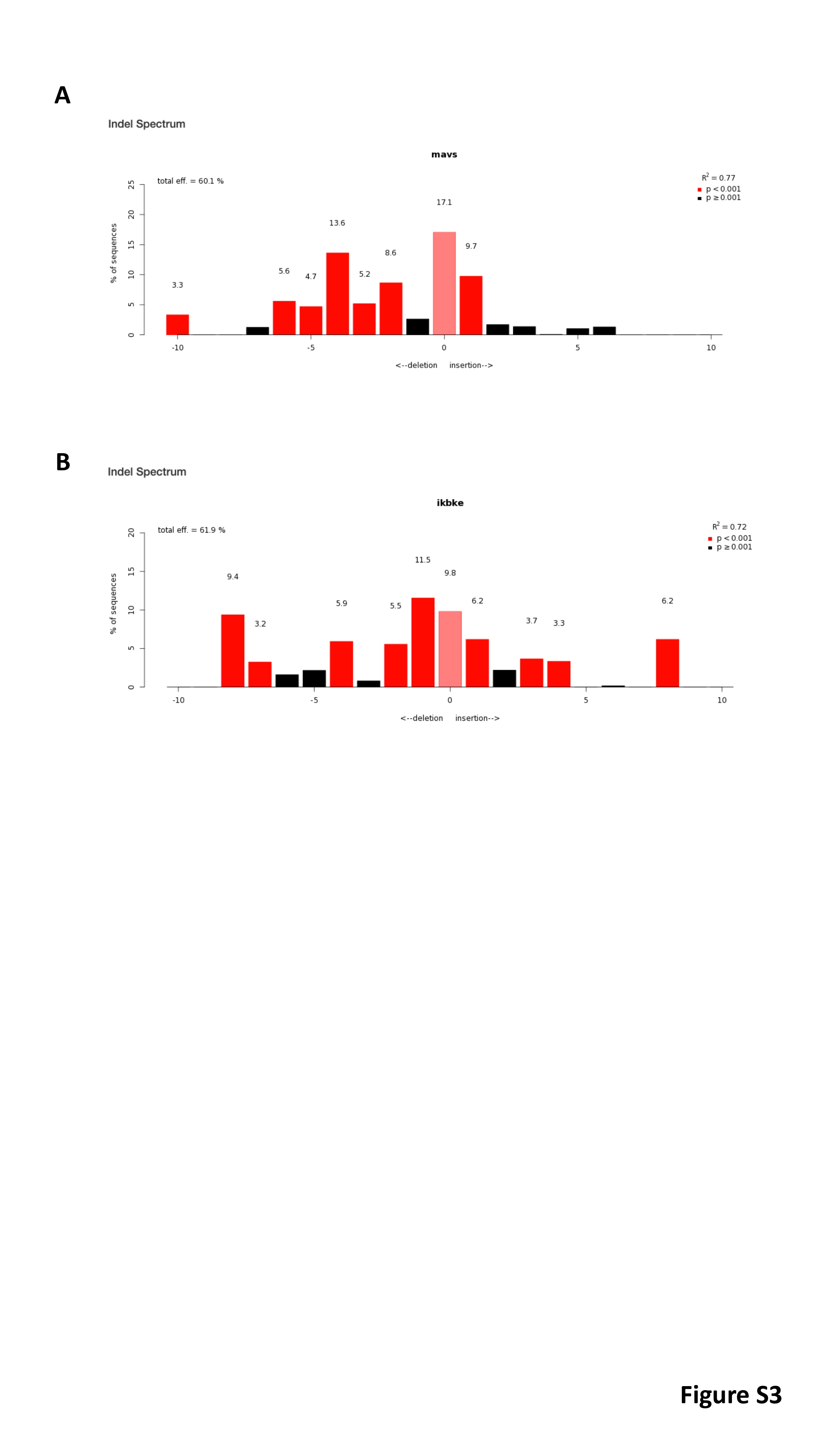

Supplement: Supplementary Figure 3 — Analysis of the efficiency of CRISPR/Cas9 edition. The efficiency of each crRNA was checked by amplifying the target sequence with a specific pair of primers (see Table S1 ) and the TIDE webtool (https://tide.nki.nl/). (A) mavs and (B) ikbke indel spectrum. [file Image_3.jpeg]

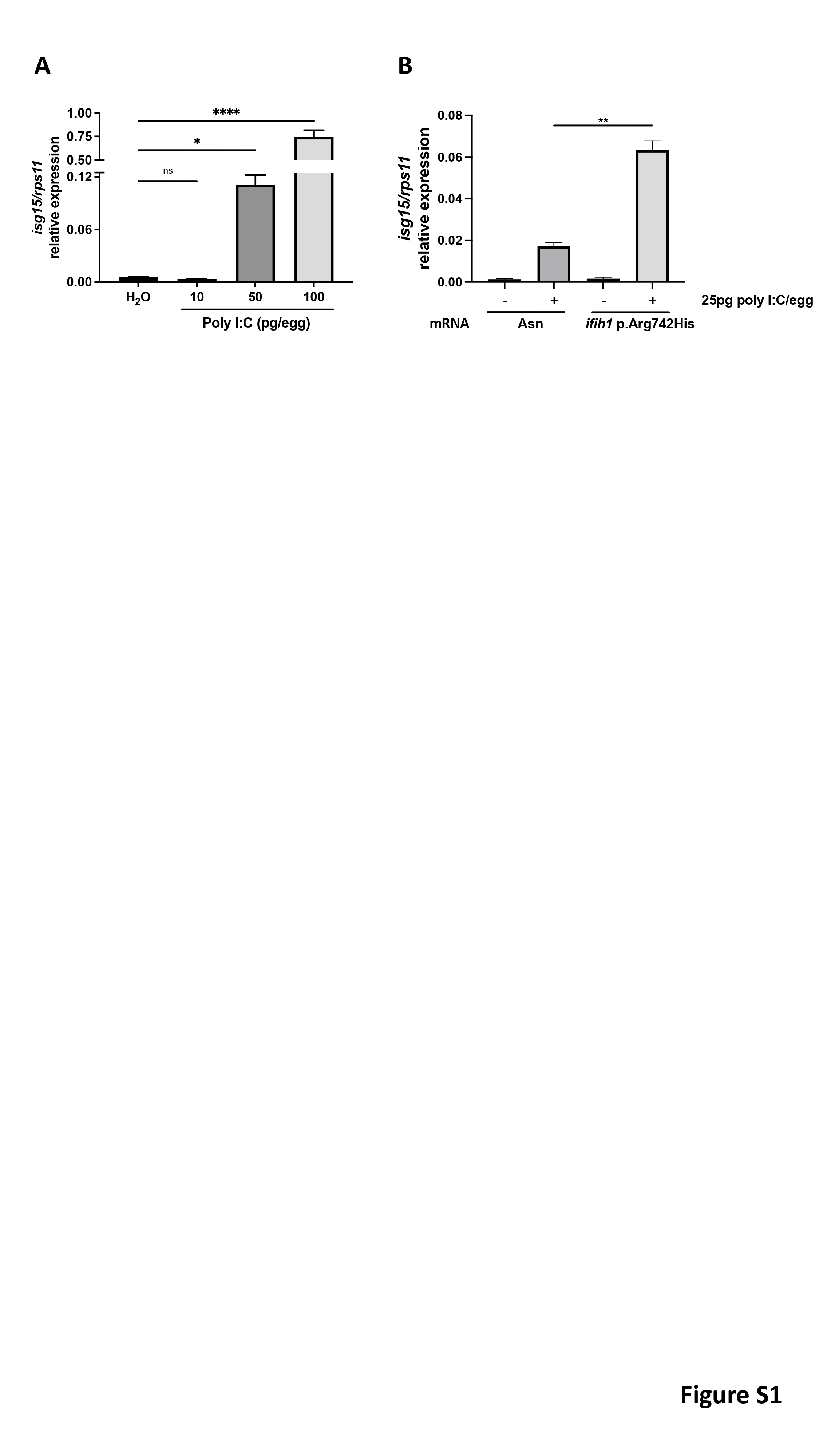

Supplement: Supplementary file 4 [file Image_4.jpeg]

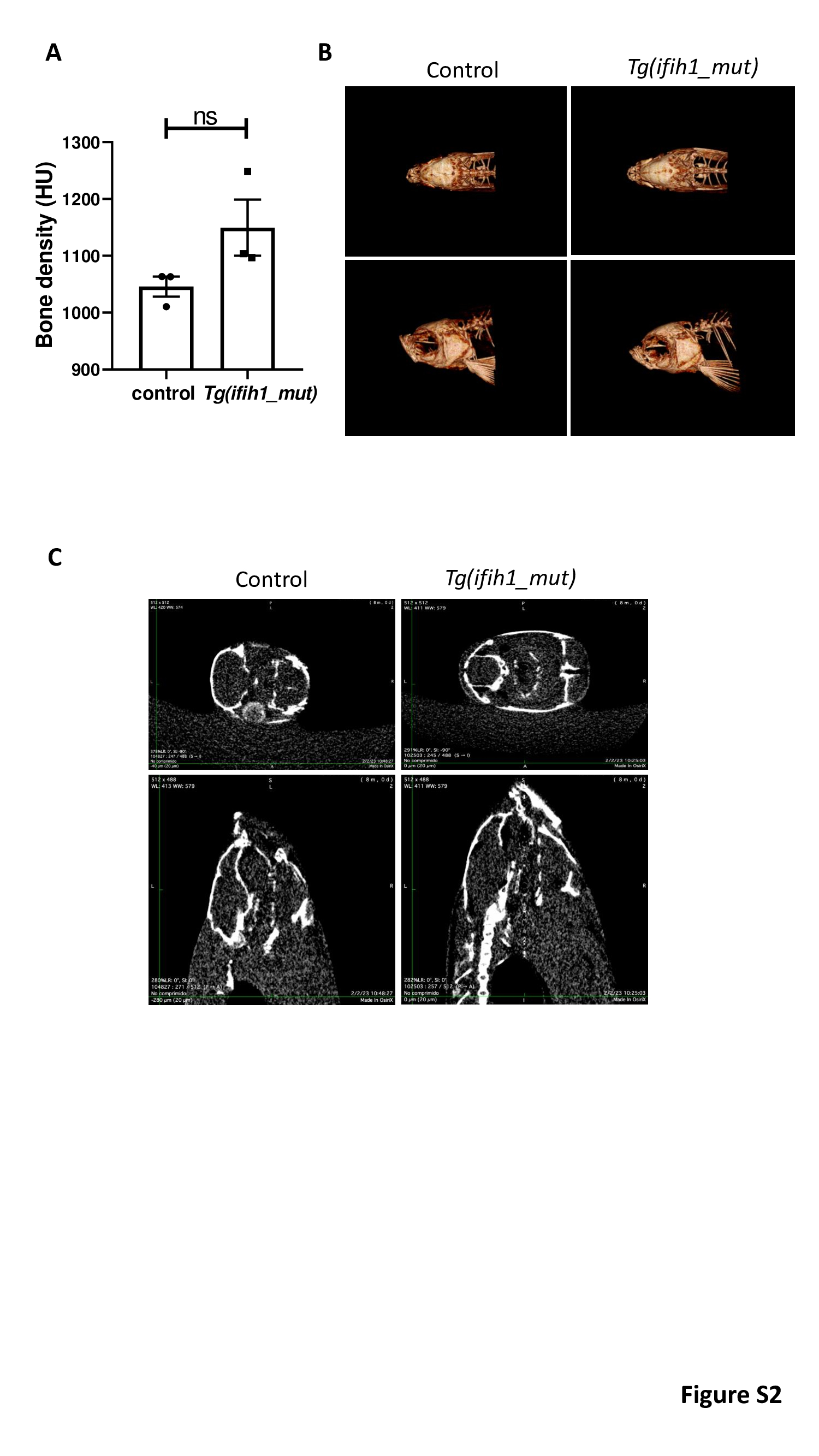

Supplement: Supplementary file 5 [file Image_5.jpeg]
